# Resected pancreatic ductal adenocarcinomas with recurrence limited in lung have a significantly better prognosis than those with other recurrence patterns

**DOI:** 10.18632/oncotarget.5054

**Published:** 2015-09-10

**Authors:** Tamna Wangjam, Zhe Zhang, Xian Chong Zhou, Laxmi Lyer, Farzana Faisal, Kevin C. Soares, Elliott Fishman, Ralph H. Hruban, Joseph M. Herman, Daniel Laheru, Matthew Weiss, Min Li, Ana De Jesus-Acosta, Christopher L. Wolfgang, Lei Zheng

**Affiliations:** ^1^ Department of Oncology, Johns Hopkins University School of Medicine, Baltimore, MD, USA; ^2^ Department of Surgery, Johns Hopkins University School of Medicine, Baltimore, MD, USA; ^3^ Department of Pathology, Johns Hopkins University School of Medicine, Baltimore, MD, USA; ^4^ Department of Radiology, Johns Hopkins University School of Medicine, Baltimore, MD, USA; ^5^ Department of Radiation Oncology, Johns Hopkins University School of Medicine, Baltimore, MD, USA; ^6^ The Sidney Kimmel Comprehensive Cancer Center, Johns Hopkins University School of Medicine, Baltimore, MD, USA; ^7^ The Skip Viragh Center for Pancreatic Cancer, Johns Hopkins University School of Medicine, Baltimore, MD, USA; ^8^ The Sol Goldman Pancreatic Cancer Center, Johns Hopkins University School of Medicine, Baltimore, MD, USA; ^9^ Division of Hematology and Oncology, University of Texas Health Science Center, San Antonio, TX, USA; ^10^ Division of Hematology and Oncology, University of Maryland School of Medicine, Baltimore, MD, USA; ^11^ University of Oklahoma Health Science Center, Oklahoma City, OK, USA

**Keywords:** pancreatic cancer, recurrent pattern, lung metastasis, prognosis

## Abstract

The majority of patients with curative resection of pancreatic ductal adenocarcinoma recur within 5 years of resection. However, the prognosis associated with different patterns of recurrence has not been well studied. A retrospective review of patients who underwent curative surgical resection of pancreatic cancer was performed. Of the 209 patients, 174 patients developed recurrent disease. Of these 174, 28(16.1%) had recurrent disease limited to lung metastases, 20(11.5%) had recurrence in the lung plus one or more other sites excluding the liver, 73(42.0%) had liver metastasis alone or liver metastasis with any other site except lung, 28(16.1%) local recurrence only, and 25(14.3%) peritoneal recurrence alone or together with local recurrence. Patients with recurrence limited to lung had a 8.5 months(Mo) median survival from recurrence to death, which was significantly better than the survival associated with recurrence in the liver(5.1Mo), in the peritoneum(2.3Mo) or locally(5.1Mo) in multivariable analyses. Among all groups, the time from surgery to the diagnosis of recurrence in patients who recurred in only in the lung was also the longest. However, 75% of patients were found to have indeterminate lung nodules on their surveillance CT scans prior to the diagnosis of recurrence in lung. This delayed diagnosis of lung recurrence may have a negative impact on survival after recurrence. In conclusion, pancreatic cancer with lung recurrence has a significantly better prognosis than recurrence in other sites. Further studies are needed to investigate how different diagnostic and treatment modalities affect the survival of this unique subpopulation of pancreatic cancer patients.

## INTRODUCTION

Pancreatic ductal adenocarcinoma (PDAC) continues to carry a very grim prognosis [1]. Even though complete surgical resection provides the only chance for cure, less than 20% of pancreatic cancer patients have surgically resectable disease at the time of diagnosis and only an additional 10% of patients become candidates for curative surgery following neoadjuvant treatment [1]. Approximately 80% of surgically resected pancreatic cancers recur within 5 years of resection, and over 60% of patients develop recurrences within 2 years [2]. The patterns of recurrence following curative surgery, and the prognosis for patients with different patterns of recurrence, have not been well characterized [3–6].

Patients with isolated recurrences in the lung have not been comprehensively analyzed although surgical resection of solitary PDAC lung metastasis was found to be potentially beneficial [7]. Thus, in this present study, we have investigated the patterns of recurrence following surgical resection of pancreatic cancer and found that recurrences limited in lung are associated with a significantly better prognosis than are other recurrence patterns. A better understanding of the patterns of recurrence may provide important insight into developing new diagnostic and management strategies for different recurrent diseases.

## RESULTS

### Recurrence limited to lung occurs in a higher frequency than historically thought

Between 1998 and 2007, 209 patients met all the eligibility criteria for this retrospective study including having surgical resection of PDAC, having postoperative follow-up primarily at the Johns Hopkins Hospital, and having archived tumor issues available for future biologic analysis (Table 1). The mean age at the time of surgery was 64.2 ± 10.9 years old. Most tumors were stage II (91.8%), with nodal involvement (86.1%), and had perineural invasion (94.1%). About half (47.8%) of the cases were found to have positive resection margins. The majority of the patients received adjuvant radiation (86.2%) and/or chemotherapy (89.2%). With a median follow-up of 16 months (range 0.8–142.9), 174 (83.3%) of the 209 patients have developed recurrent disease.

**Table 1:** Baseline characteristics of all 209 patients, patients with all types of recurrence and patients with lung recurrence

Variables	All patients (*n* = 209)	Patients with all types of recurrence (*n* = 174)	Patients with lung recurrence (*n* = 24)
	mean(±SD)\medain(range)\n(%)	mean(±SD)\median(range)\n(%)	mean(±SD)\median(range)\n(%)
Age (years)	64.2(±10.9; 30–84)	63.7(±10.8; 30–84)	65.2(±8.5; 47–80)
Gender (male)	109(52.2%)	86(49.4%)	15(53.6%)
Stage			
I	11(5.3%)	10(5.7%)	1(3.6%)
II	191(91.8%)	160(92%)	27(96.4%)
III	6(2.9%)	4(2.3%)	0(0%)
Positive lymph nodes	180(86.1%)	151(86.8%)	26(92.9%)
Tumor size (diameter; cm)	3.2(±1.3; 1–7.9)	3.2(±1.3; 1–7.9)	2.9(±1.2; 1–5.3)
Positive margins	100(47.8%)	80(46%)	15(53.6%)
Tumor grade/differentiation			
I/Well	4(1.9%)	4(2.3%)	0(0%)
II/Moderate	108(51.9%)	95(54.6%)	21(75%)
III/Poor	96(46.2%)	75(43.1%)	7(25%)
Vascular Invasion	103(56%)	84(56%)	13(50%)
Perineural Invasion	192(94.1%)	160(94.1%)	24(88.9%)
Adjuvant Radiation	156(86.2%)	136(85.5%)	24(88.9%)
Adjuvant Chemo	165(89.2%)	146(89%)	26(96.3%)

The most common site of the first recurrence for the 174 patients was the liver (alone or in combination with any other sites except lung), seen in 73 (42.0%) cases (Table 2). The remaining recurrences were more equally distributed: 28 (16.1%) had recurrence limited to lung only, 20 (11.5%) had recurrence in lung with one or more sites except liver, 28 (16.1%) had local recurrence only, and 25 (14.4%) had peritoneal recurrence alone or with local recurrence. It should be noted that the prevalence of recurrence limited to lung or in lung together with one or more sites outside the liver was much higher than those reported historically [3]. This new finding raised our interests in further characterizing this patient population.

**Table 2:** Clinical outcomes between different recurrence patterns for patients with recurrences

Recurrence location	Number (Percent)	Median OS (months) (95% CI)	Median STR (months) (95% CI)	Median RTD (months) (95% CI)
All recurrences	174 (100%)	18.0(15.3, 19.5)	10.1(9.1- 11.3)	5.1(4.8–6.5)
Lung only	28 (16.1%)	27.8 (18.2–50.0)	12.7 (9.9–30.8)	8.5 (5.7–19.5)
Lung with any other site	20 (11.5%)	19.0 (14.6- 49)	11.4 (8.0- 25.7)	9.4 (4.9–23.2)
Liver (alone or with any other site except lung)	73 (42.0%)	16.6 (12.4–19.5)	8.9 (7.3–10.4)	5.1 (4.3–7.5)
Peritoneal (including malignant ascites, ovarian metastasis)	25 (14.4%)	13.6 (10.2–19.5)	10.6 (7.3–16.2)	2.3 (1.8–4.0)
Local only	28 (16.1%)	15.1 (13.8–28.6)	10.4 (6.7–17.5)	5.1 (2.6–7.5)

OS = overall survival/time from surgery to death; STR = time from surgery to diagnosis of recurrence; RTD = time from recurrence to death; CI = confidence interval

### Time from surgery to death among all recurrence patterns

The median overall survival (OS) for all of the 209 patients, which was calculated with the time from surgery to death was 17.5 months (95% CI 14.9–19.3). For the patients with recurrent disease limited to the lung, the median survival time was found to be 27.8 months (95% CI 18.2–50). The median survival times associated with the other patterns of recurrence are presented in Table 2.

We also performed univariate and multivariable analyses of OS together with potential confounding factors. In this univariate analysis of OS, higher tumor T stage (HR 5.39, 95% CI 1.42–20.45, *p* = 0.01), higher tumor grade (HR 1.39, 95% CI 1.01- 1.89, *p* = 0.04), presence of perineural invasion (HR 4.43, 95% CI 1.91–10.28, *p* = 0.001), positive resection margins (HR 1.66, 95% CI 1.21–2.28, *p* = 0.002), and lack of adjuvant radiation (HR 1.64, 95% CI 1.03- 2.63, *p* = 0.04) were all found to be associated with worse survival outcome (Table S1).

### Lung recurrence was associated with the longest median survival time following recurrence among all recurrence patterns

**Figure 1:**
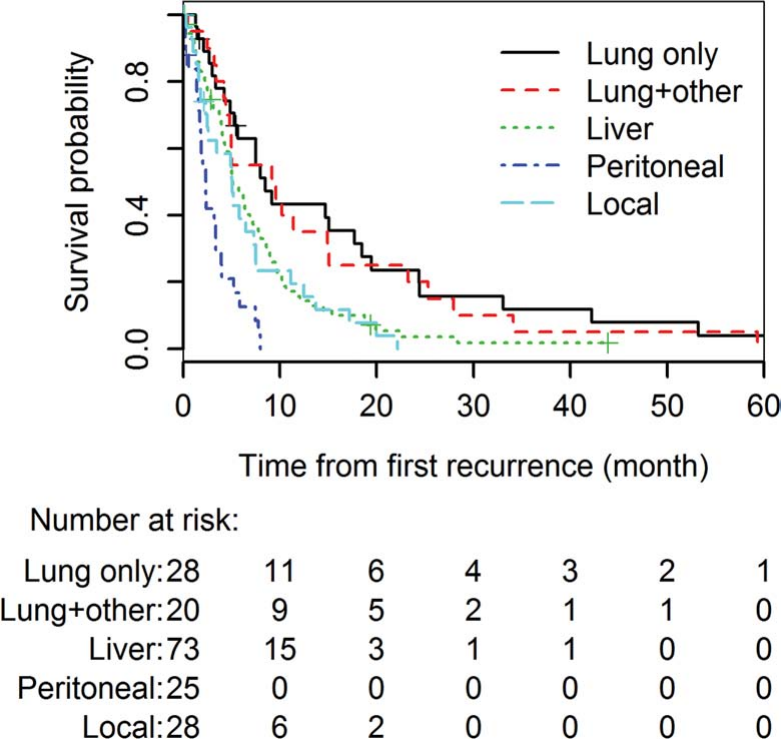
Kaplan-Meier analysis of the survival times (RTD) after different types of recurrence following surgical resection of pancreatic cancer

Next, we examined the time from the first recurrence to death (RTD) (Table 2 and Figure 1). The median RTD survival time for all the 174 patients with recurrent disease was 5.1 months (95% CI: 4.8–6.5). Patients with lung recurrence had a median RTD of 8.5 months (95% CI: 5.7–19.5). Univariate analysis (Table S2) of the RTD shows that positive margins (HR 1.37, 95% CI: 1.01–1.88, *p* = 0.046), presence of perineural invasion (HR 2.82, 95% CI: 1.29–6.20, *p* = 0.010) and shorter time from surgery to recurrence (STR) (HR 0.98, 95% CI: 0.97–1.00, *p* = 0.011) were associated with a shorter RTD. Recurrence in liver (with and without other sites) (HR 1.93 95% CI: 1.20 - 3.11, *p* = 0.006), peritoneal recurrence (HR 5.42, 95% CI: 2.96 - 9.91, *p* < 0.001) and local recurrence (HR 2.25, 95% CI: 1.28 – 3.97, *p* = 0.005) all were associated with a significantly unfavorable RTD comparing to lung recurrence only. The association of lung recurrence with longer RTD was also independent from other known prognostic factors in the multivariate analysis (Table 3). This result again suggests that lung recurrence is associated with a better survival following the identification of recurrent disease than are other patterns of recurrence.

**Table 3:** Multivariable analysis for time from recurrence to death

Variable	HR(95% CI)	*P* value
Age	1.00 (0.98, 1.02)	0.874
Gender (female vs. male)	0.89 (0.60, 1.31)	0.544
Tumor size	1.13 (0.97, 1.33)	0.127
Tumor grade		
Grade II vs. I	1.00 (0.69, 1.45)	0.991
Grade III vs. I	0.93 (0.27, 3.17)	0.906
Lymph Nodes (yes vs. no)	1.39 (0.81, 2.38)	0.229
Margin (positive vs. negative)	1.54 (1.06, 2.25)	0.025
Vascular invasion (yes vs. no)	0.99 (0.69, 1.42)	0.967
Perineural invasion (yes vs. no)	2.19 (0.87, 5.49)	0.097
Time from surgery to first recurrence	1.00 (0.98, 1.02)	0.883
Recurrence pattern		
Lung + Other vs. Lung	1.25 (0.63, 2.48)	0.516
Liver + Other vs. Lung	1.91 (1.10, 3.31)	0.022
Peritoneal vs. Lung	6.32 (3.12, 12.79)	< .001
Local vs. Lung	2.09 (1.10, 4.00)	0.025

HR = hazard ratio; CI = confidence interval

### Time from surgery to the diagnosis of recurrence

The above results suggest that the survival time after lung recurrence is independent of the time from surgery to the diagnosis of recurrence (STR) (Table 3). The median STR of patients with any recurrence was 10.1 months (range: 9.0–11.1) (Table 2). The STR in patients who recurred in their lungs only was 12.7 months (range: 9.9–30.8) (Table 2). In a univariate analyses for STR, it was observed that positive tumor margins, higher tumor stage (stage II > I), presence of lymph nodes, higher tumor grade (poorly/grade III and moderately/grade II vs. well differentiated/grade I), presence of perineural invasion, lack of adjuvant radiation and adjuvant chemotherapy, were associated significantly with reduced STR (Table S3).

### Delayed diagnosis of lung recurrence

We found that, out of the 28 patients with lung recurrence alone, 24 presented with lung nodules on CT scans while 4 had malignant pleural effusions, without any other sites of recurrence. Out of the 24 patients with lung nodules, 18 (75%) had indeterminate lung nodules in their surveillance CT scans prior to the definitive diagnosis of lung recurrence. The median time from the surgery to the initial presentation of lung nodules (STN) was as short as 10.2 Mo (range: −0.9, 48.10). It should be noted that two patients with subsequent diagnosis of lung metastasis presented with indeterminate lung nodules prior to the surgical resection of primary pancreatic tumors. Thus, our study suggested that there was a delay in the diagnosis of lung recurrence even though the prognosis of lung recurrence was better than any other recurrence pattern.

### Delayed diagnosis of lung recurrence has an impact on survival after recurrence

Next, we took a close examination of the patients with lung recurrence. Their median age was 65.2 yrs (range: 47–80) and 53.6% were males. The median diameter of their primary pancreatic tumor was 2.9 cm (range:1–5.3). Most of the patients had positive margins (53.6%), stage II diseases (96.4%), positive lymph nodes (92.9%), had moderately differentiated (75%) cancers, and most had perineural invasion (88.9%). Half the cases had vascular invasion. The majority of the patients received adjuvant radiation (88.9%) and chemotherapy (96.3%) (Table 1). The clinicopathologic characters of this subgroup of patients are quite comparative to those with other patterns of recurrence (Table 1 and Table S4) and a univariate analysis did not identify any association between the clinicopathologic characters of this subgroup and RTD (Table S5). Among the 24 patients who had lung nodules detected, the multivariable analysis, after adjusting for age, margin, lymph node status and treatment for lung recurrence, suggested a delay in the diagnosis of lung nodules as lung recurrences was associated with a shorter RTD (HR = 4.51, 95% CI = 1.27 - 6.1, *p* = 0.020) (Table 4). Therefore, our data suggest that a delay in the diagnosis of lung nodules as recurrence may have a negative impact on survival after recurrence.

**Table 4:** Multivariable analysis for the association between delayed diagnosis of lung nodules as recurrence and RTD (*n* = 24)*

Variable	HR(95% CI)	*p*-value
Delayed diagnosis of lung nodules as recurrence (yes vs. no)	4.51 (1.27, 16.06)	0.020
Age	1.04 (0.95, 1.13)	0.400
Margin (positive vs. negative)	0.53 (0.19, 1.45)	0.220
Lymph nodes (yes vs. no)	9.69 (0.87, 108.5)	0.065
Treatment after lung recurrence (Yes vs. no)	0.71 (0.27, 1.92)	0.510

HR = hazard ratio; CI = confidence interval

After lung recurrence, approximately 67% of the patients received one or more anti-cancer treatments including surgical resection of oligometastases, chemotherapy and radiation therapy. However, our data did not suggest that patients who received the treatments had significantly different RTD from those who did not receive any treatment (HR = 0.71, 95% CI = 0.27–1.92, *p* = 0.510) (Table 4).

## DISCUSSION

In this study, we demonstrate that the incidence of recurrence in the lung following surgical resection of pancreatic cancer is much higher than was thought historically [3]. The prognostic analysis of clinicopathologic factors in this study patient population is consistent with previous reports on the prognostic factors following surgical resection of pancreatic cancer [8, 9]. We also demonstrate that pancreatic cancer with the first recurrence only in the lung is associated with a significantly better prognosis following recurrence than are recurrences in other sites. In addition, the diagnosis of lung recurrence appeared to be often delayed from the time of first appearance of lung nodules.

In the past, the incidence of recurrence in the lung was considered to be as low as 1–2% [3]. In a more recent study, the incidence was reported to be 13.64%, likely due to the more frequent use of CT scanning of the chest as part of surveillance [5]. Our study demonstrated a similar incidence of recurrence in the lung (11.5% lung only recurrence). Although we cannot pathologically exclude that some of these patients had a second primary lung adenocarcinoma, such events in the period of time over which the patients were observed would be rare. It should be noted that in our study we found that patients with a first recurrence in lung and at least one additional recurrent site had a similar good prognosis compared to patients with the first recurrence only in the lung. Certainly and very likely, the patients with lung and at least one additional recurrent site at the time of diagnosis of recurrence may have developed the recurrence in lung as indeterminate lung nodules before they developed the recurrences in the other sites; thus, their prognosis is similar to those who had only the recurrence in lung at the time of diagnosis. These results suggest that patients with lung recurrence have a better prognosis, not necessarily due to the restriction of metastatic diseases to lung, but more likely due to the underlying biologic mechanisms that determine the propensity of first recurrence site. It should be also noted that lung recurrence has a better outcome that local recurrence, further suggesting that the underlying biology may have determined the outcomes of different recurrence patterns. It will be intriguing to investigate specific biologic pathways that have determined each individual pattern of recurrence. Understanding the biology behind the prognostic difference in different recurrence patterns may facilitate the development of new therapeutic strategies to further enhance the survival of patients who have a potential of developing lung metastases or to reverse the poor outcome of those who have a potential of developing metastases at other organ sites.

We also found that diagnosis of lung recurrence was usually delayed and the data suggest a statistically significant, negative impact of this delay on survival time following disease recurrence in the multivariable analysis but not in the univariate analysis. The difference between multivariate and univariate analyses could likely be due to the lack of statistical power as well as the presence of interaction between the variables included in the multivariate model, which should be investigated in a future larger cohort. It should also be noted that the calculation of RTD would start from a later time point for those patients whose diagnosis of lung recurrence was delayed, which may lead to the shortening of RTD in the patients with delayed diagnosis of lung recurrence.

Nevertheless, whether the patients receive anti-cancer treatments following the diagnosis of lung recurrence does not appear to have an impact on the RTD. Potential explanations include that the patients who had an indolent disease tended to decline any treatment and that the benefit of the current treatments may be too small to be demonstrated in a small-size patient population. It is also possible that those patients who could have benefited from the treatment had a delayed diagnosis and thus a delayed initiation of treatment. Our published study of a different patient population showed that selected patients who underwent thoracic metastectomy following lung recurrence had a long-term survival, suggesting that selected patients with lung recurrence may benefit from the treatment [7]. Therefore, it is possible that early diagnosis of lung recurrence may help select better candidates for metastectomy. However, whether treatments will affect the outcome of lung recurrence should be further investigated in a prospective study.

It would be particularly interesting to investigate the impact of immune-based therapy that may affect the survival of pancreatic cancer patients with different metastatic or recurrence patterns. Because different organs provide different tumor microenvironment (TME) and are associated with their unique inflammatory processes at different levels, immune based therapies targeting TME may have different impacts on treating different recurrent diseases. Lung metastases may be more susceptible to immune based therapy, considering that lung is naturally and abundantly infiltrated with immune cells due to constant exposure to potential pathogens [10].

This study was limited due to the small sample size of patients in individual subgroups of recurrence patterns, from the inclusion of patients only from a single institution, and the lack of comprehensive data from outside facilities where some of patients were also being followed in addition to being followed at the Johns Hopkins Hospital. There were no standardized criteria for the diagnosis of recurrent disease and was left to the discretion of the treating clinicians. For example, of the 28 patients who were diagnosed with lung recurrence alone, approximately 20% were diagnosed radiographically although all their following clinical courses were consistent with the presence of lung metastases. Nevertheless, the association of better prognosis with lung only recurrence was confirmed in our preliminary report of a larger cohort of 1,138 consecutive patients with PDAC who underwent pancreatectomy at the Johns Hopkins Hospital [11].

The findings of this study have the potential in helping understand the biology of recurrent disease and may help guide follow-up and therapy for recurrent disease. It will be also intriguing to investigate the causes that the patients with different recurrence pattern died of. Future multi-center studies are needed to confirm these findings and to investigate further how different diagnostic and treatment modalities can improve the survival for patients with recurrence following the surgical resection of pancreatic cancer. This study also highlights the need to identify tumor characteristics and biomarkers that bestow the favorable prognosis of lung recurrence. Since recurrent disease in the lung has a better prognosis compared to other sites, early diagnosis of lung nodules as metastases, more aggressive interventions including surgical resection of oligometastases, and more effective therapeutics to be developed to target the mechanisms underlying the lung metastasis process may have a great impact on overall survival and thus warrant further investigation in both retrospective and prospective studies.

## MATERIALS AND METHODS

### Patients

After institutional review board approval, a retrospective chart review was conducted on a total of 209 consecutive patients who had curative surgical resection of pancreatic adenocarcinomas from January 9th, 1998 to June 13th, 2007, had archived tissues available for future biologic analyses, and had postoperative follow-up primarily at the Johns Hopkins Hospital. All the data pertaining to the patient's demographics, medical history and follow-up were collected from the electronic medical records. Survival and cancer-specific deaths were determined by review of clinical follow-up information, Social Security Death Index and the National Cancer Database. Patients were followed till death or last day known to be alive. All pertinent charts including survival information dated on or prior to July 17th 2012 were reviewed.

Data on the timing of recurrence and recurrence patterns were collected. Patients who had the first recurrence in the lung were analyzed in detail through reviewing electronic medical record to gather demographic, clinical, laboratory test, pathological, imaging, treatment and disease recurrence information. Disease recurrence was determined from the medical record, either clinically by imaging studies (computed tomography, positron emission tomography), or by tissue diagnosis (CT-guided biopsy, wedge resection or lobectomy).

Pleural effusion without parenchymal disease or without malignant cytological findings was not considered to be lung metastasis. However, cytologically confirmed malignant isolated pleural effusion with or without biopsy proven malignant lung nodules were considered lung recurrences. Cytologically proven peritoneal effusion without peritoneal metastases and recurrent disease in the ovaries and adrenals were considered as peritoneal recurrence.

### Statistical analysis

The primary outcome of interest was survival after disease recurrence (RTD), which was defined as the time from diagnosis of recurrence to the time of death. Those remained alive without documentation of death were censored at the time of their last follow-up. Survival distributions of different patterns of recurrence were described using the Kaplan-Meier method and compared using the log-rank test. Hazard ratios were estimated by the Cox proportional hazards model. Since patients included in this analysis had all recurred, time to disease recurrence was considered as a continuous variable and adjusted for along with other clinical risk factors in the multivariable regression model. The time delayed from the initial detection of lung nodules on CT scan of the chest to the diagnosis of lung metastasis, designated nodule latency, was calculated from the time when any pulmonary nodule was first noticed radiographically to the time when they were diagnosed as being recurrent PDAC in the lung. Potential impact of delayed diagnosis in lung recurrence on RTD was evaluated using Cox models as well, in which delayed diagnosis in lung recurrence was considered as a dichotomized baseline covariate. Other clinical outcomes including time from surgical resection of primary disease to diagnosis of recurrent disease (STR) and time from surgery to death/overall survival (OS) were analyzed in a descriptive manner. All tests were two-sided and considered statistically significant at *P* < 0.05. Statistical analyses were performed using SAS (version 9.3, SAS Institute, Cary, NC) and R statistical software (version 2.15.2).

## SUPPLEMENTARY TABLES


