# Supplementary material for: Resected pancreatic ductal adenocarcinomas with recurrence limited in lung have a significantly better prognosis than those with other recurrence patterns
Source: Oncotarget. 2015 Sep 10;6(34):36903–10. doi: 10.18632/oncotarget.5054 (PMC4742219; doi:10.18632/oncotarget.5054)
Supplement: Supplementary file 1 [file oncotarget-06-36903-s001.pdf]

SUPPLEMENTARY TABLES

Supplementary Table S1: Univariate analysis for OS of all types of recurrence (*n* = 174)

Variable	OS	
	**HR(95% CI)**	***P*-value**
Age	0.99(0.98, 1.01)	0.38
Gender(female vs. male)	0.9(0.66, 1.22)	0.49
Stage		
II vs. I	1.74(0.88, 3.44)	0.11
III vs. I	5.39(1.42, 20.45)	0.01
Lymph nodes (yes vs. no)	1.54(0.98, 2.42)	0.06
Margin (positive vs. negative)	1.66(1.21, 2.28)	0.002
Grade		
II vs. I	0.72(0.53, 0.99)	0.04
III vs. I	0.38(0.14, 1.05)	0.06
Vascular Invasion (yes vs. no)	0.98(0.7, 1.37)	0.89
Perineural Invasion (yes vs. no)	4.43(1.91, 10.28)	0.001
Adjuvant Radiation (yes vs. no)	0.61(0.38, 0.97)	0.04
Adjuvant Chemo (yes vs. no)	0.63(0.37, 1.07)	0.09

HR = hazard ratio; CI = confidence interval

Supplementary Table S2: Univariate analysis for RTD of all types of recurrence (n = 174)

Variables	HR(95% CI)	*P*-value
Age	0.99(0.98, 1.01)	0.35
Gender(male)	0.92(0.67, 1.25)	0.58
Margin (positive vs. negative)	1.37 (1.01, 1.88)	0.05
Tumor size	1.1(0.98, 1.24)	0.10
Stage		
II vs. I	1.34(0.68, 2.65)	0.40
III vs. I	2.45(0.65, 9.22)	0.19
Lymph nodes	1.29(0.82, 2.03)	0.27
Grade		
II vs. III	0.91(0.66, 1.24)	0.54
I vs. III	0.49(0.18, 1.33)	0.16
Vascular Invasion	1.01(0.72, 1.42)	0.94
Perineural Invasion	2.82(1.29, 6.2)	0.01
Adjuvant Radiation	1.27(0.8, 2.02)	0.31
Adjuvant Chemo	1.11(0.66, 1.86)	0.70
Time from surgery to recurrence	0.98(0.97, 1.00)	0.01
Recurrence site		
Lung + other vs. Lung only	1.12(0.62, 2.02)	0.70
Liver vs. Lung only	1.93(1.20, 3.11)	0.006
Peritoneal vs. Lung only	5.42(2.96, 9.91)	< 0.001
Local vs. Lung only	2.25(1.28, 3.97)	0.005

HR = hazard ratio; CI = confidence interval

Supplementary Table S3: Univariate analysis for STR of all types of recurrence (*n* = 174)

Variable	HR(95% CI)	*P*-value
Age	0.99(0.98, 1.01)	0.32
Gender(male)	0.9(0.67, 1.22)	0.50
Margin (positive vs. negative)	1.8(1.31, 2.46)	< 0.001
Tumor size	1.3(46.98, 1.9)	0.92
Stage		
II vs. I	1.64(0.86, 3.12)	0.13
III vs. I	4.1(1.26, 13.28)	0.02
Lymph node (yes vs. no)	1.56(1, 2.43)	0.05
Grade		
II vs. III	0.68(0.5, 0.92)	0.01
I vs. III	0.36(0.13, 0.98)	0.05
Vascular Invasion (yes vs. no)	1.03(0.74, 1.43)	0.86
Perineural Invasion(yes vs. no)	2.95(1.48, 5.88)	0.002
Adjuvant Radiation(yes vs. no)	0.24(0.15, 0.38)	< 0.001
Adjuvant Chemo(yes vs. no)	0.4(0.24, 0.66)	< 0.001

HR = hazard ratio; CI = confidence interval

Supplementary Table S4: Comparison of baseline characteristics by recurrence pattern

Variables	No recur	Lung	Lung + other	Liver + other	Peritoneal	Local	*P*-value*
Age, Years	67(±11.6)	65.2(±8.5)	68(±9.9)	63.6(±11)	60.4(±12.9)	62.2(±10.1)	0.152
Gender							
Male	23	15	7	37	12	15	
Female	12	13	13	36	13	13	0.717
Positive margins							
Yes	20	15	8	30	12	15	
No	15	13	12	43	13	13	0.683
Stage							
I	1	1	2	3	3	1	
II	31	27	16	69	22	26	
III	2	0	2	1	0	1	0.212
Lymph nodes							
Yes	29	26	17	65	18	25	
No	6	2	3	8	7	3	0.185
Tumor size	3.3(±1.1)	2.9(±1.2)	3.6(±1.7)	3.2(±1.2)	3.1(±1.3)	3.3(±1.2)	0.447
Grade							
Well	0	0	2	2	0	0	
Moderate	13	21	10	31	17	16	
Poor	21	7	8	40	8	12	0.027
Vascular Invasion							
Yes	19	13	12	31	10	18	
No	15	13	6	27	12	8	0.390
Perineural Invasion							
Yes	32	24	16	69	24	27	
No	2	3	2	3	1	1	0.556
Adjuvant Radiation							
Yes	20	24	13	56	22	21	
No	2	3	7	12	0	1	0.011
Adjuvant Chemo							
Yes	19	26	19	57	21	23	
No	2	1	1	11	2	3	0.389

*The *p*-value only pertains to groups with recurrences. The group with no recurrence was not included for comparison. Comparisions were performed using ANOVA for continuous variables and Exact Pearson Chi square test for categorical variables.

Supplementary Table S5: Univariate analysis for the association between delayed diagnosis of lung nodules as recurrence and RTD (*n* = 24)

Variable	HR(95% CI)	*P*-value
Age	0.98(0.91, 1.04)	0.448
Gender (male vs. female)	0.84(0.38, 1.85)	0.659
Margin (positive vs. negative)	0.93(0.42, 2.05)	0.850
Tumor size	1.05(0.74, 1.51)	0.779
Lymph nodes (yes vs. no)	4.07(0.53, 31.29)	0.177
Grade		
II vs. III	1.21(0.49, 2.97)	0.681
II vs. III	-	-
Vascular Invasion (yes vs. no)	0.78(0.34, 1.78)	0.556
Perineural Invasion (yes vs. no)	2.1(0.48, 9.27)	0.325
Adjuvant Radiation (yes vs. no)	2.42(0.68, 8.55)	0.171
Adjuvant Chemo (yes vs. no)	0.67(0.09, 5.18)	0.702
Time from surgery to recurrence	0.99(0.96, 1.02)	0.416
Delayed diagnosis of lung recurrence(yes vs. no)	2.02(0.77, 5.34)	0.155
Treatment after lung recurrence (Yes vs. no)	0.88 (0.39, 1.95)	0.745

HR = hazard ratio; CI = confidence interval
